# Correction: Targeted treatment of rat AKI induced by rhabdomyolysis using BMSC derived magnetic exosomes and its mechanism

**DOI:** 10.1039/d5na90072g

**Published:** 2025-10-29

**Authors:** Yuling Chen, Shike Hou

**Affiliations:** a Institute of Disaster and Emergency Medicine, Tianjin University Tianjin China chenyuling97@outlook.com; b Tianjin Key Laboratory of Disaster Medicine Technology Tianjin China

## Abstract

Correction for ‘Targeted treatment of rat AKI induced by rhabdomyolysis using BMSC derived magnetic exosomes and its mechanism’ by Yuling Chen *et al.*, *Nanoscale Adv.*, 2024, **6**, 4180–4195, https://doi.org/10.1039/D4NA00334A.

The authors regret that duplicated panels were accidently presented in Fig. 4 (control panels for IL-1β and IL-10). When looking back through the raw western blot data, the authors also noted an error in Fig. 6 panel A, where the representative images of protein imprinting in the kidneys, specifically in the INOS 131 kDa panel, IL-10 20 kDa and one of the GAPDH 36 kDa panels, were incorrect.

Raw data has been provided and the validity of the conclusions has been confirmed by an independent expert. Corrected versions of Fig. 4 and 6 are provided in this correction notice.

**Fig. 1:**
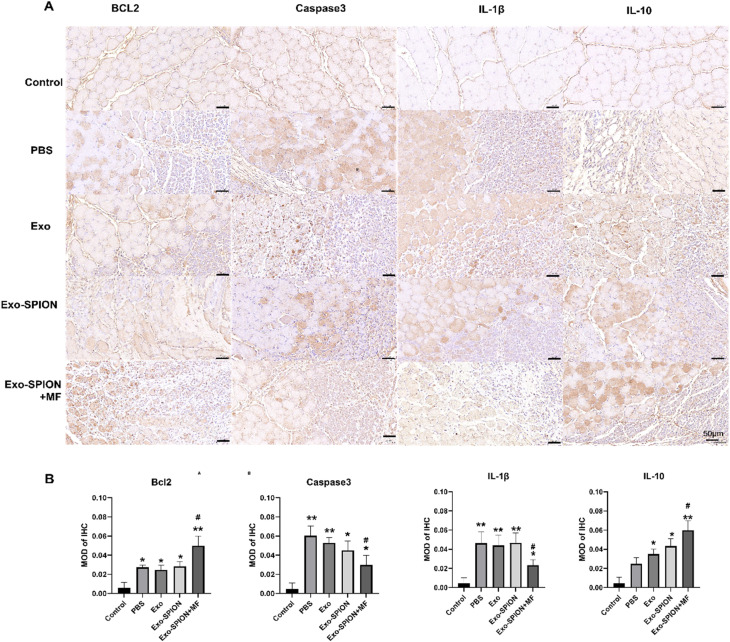
The expression of Bcl2, Caspase3, IL-1β and IL-10 positive cells in the muscles of rats in each group. (A) The expression level in the muscle tissues was showed by IHC. Scale bar, 50 μm. (B) Quantitative analysis of IL-1β, IL-10, Caspase3 and Bcl2 protein expression in muscle of rats in each group. *P < 0.05, **P < 0.01 compared to the control group. #*P* < 0.05 compared to PBS group.

**Fig. 2:**
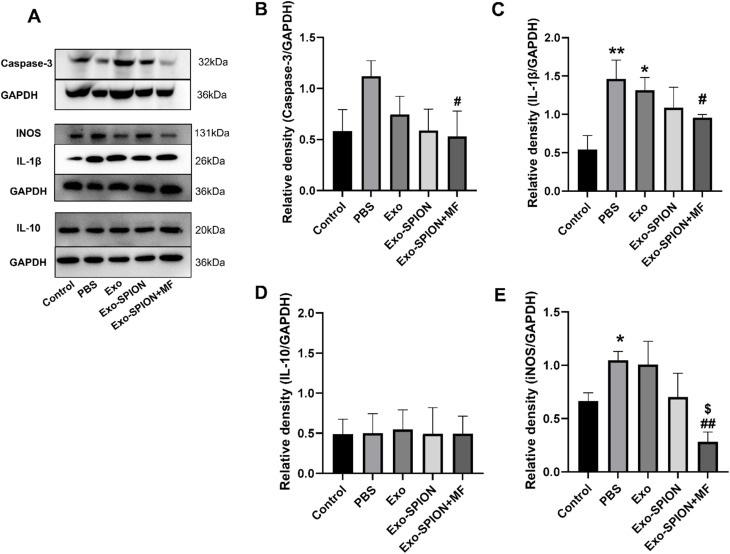
Expression of proteins in the kidneys of rats. Compared with those in the control group, the inflammatory and apoptotic factors in the kidneys of the rats significantly increased within 3 days after modeling. (A) Representative images of protein imprinting in the kidneys. (B–E) Protein blotting analysis showed that treatment with exogenous Exo-SPION + MF prevents renal inflammation and apoptosis in AKI rats. *P < 0.05, **P < 0.01, ***P < 0.001 compared to the control group. #*P* < 0.05, ##*P* < 0.01, ###*P* < 0.001 compared to the PBS group (*n* = 6).

The Royal Society of Chemistry apologises for these errors and any consequent inconvenience to authors and readers.

